# Prognostic value of ^18^F-FDG brain PET as an early indicator of neurological outcomes in a rat model of post-cardiac arrest syndrome

**DOI:** 10.1038/s41598-019-51327-1

**Published:** 2019-10-15

**Authors:** Daehee Kim, Hai-Jeon Yoon, Woon Jeong Lee, Seon Hee Woo, Bom Sahn Kim

**Affiliations:** 1 Department of Emergency Medicine, Incheon St. Mary’s Hospital, The Catholic University of Korea, Incheon, Korea; 2 Department of Nuclear Medicine, College of Medicine, Ewha Womans University, Seoul, Korea; 3 Department of Emergency Medicine, College of Medicine, The Catholic University of Korea, Seoul, Korea

**Keywords:** Predictive markers, Experimental models of disease

## Abstract

Predicting neurological outcomes in patients with post-cardiac arrest syndrome (PCAS) is crucial for identifying those who will benefit from intensive care. We evaluated the predictive value of 18F-FDG PET. PCAS was induced in Sprague Dawley rats. Baseline and post-3-hour images were acquired. Standardized uptake value (SUV) changes before and after PCAS induction (SUV_delta_) and SUV ratios (SUVR) of regional SUV normalized to the whole brain SUV were obtained. The Morris water maze (MWM) test was performed after 2 weeks to evaluate neurological outcomes and rats were classified into two groups based on the result. Of 18 PCAS rats, 8 were classified into the good outcome group. The SUV_delta_ of forebrain regions were significantly decreased in good outcome group (p < 0.05), while the SUV_delta_ of hindbrain regions were not significantly different according to outcomes. The SUVR of forebrain regions were significantly higher and the SUVR of hindbrain regions were significantly lower in good outcome group (p < 0.05). Forebrain-to-hindbrain ratio predicted a good neurological outcome with a sensitivity of 90% and specificity of 100% using an optimal cutoff value of 1.22 (AUC 0.969, p < 0.05). These results suggest the potential utility of 18F-FDG PET in the early prediction of neurological outcomes in PCAS.

## Introduction

Cardiac arrest carries an extremely high mortality rate. Mortality for out-of-hospital cardiac arrests is greater than 90%, with many survivors incurring severe neurological impairment despite improvements in resuscitation practices^1–3^. Such high rates of mortality and morbidity are largely due to brain and cardiac dysfunction, a syndrome known as post-cardiac arrest syndrome (PCAS)^4^. Prolonged intensive care of patients with severe neurological impairment carries enormous psychological and financial costs to the family. However, despite the poor results, some patients can achieve a good recovery from PCAS and a restored quality of life similar to that before cardiac arrest.

Clinicians, especially those in emergency medicine departments, are constantly required to make decisions on the provision of intensive care for PCAS patients, and proper patient stratification requires accurate methods for predicting neurological outcomes. The most commonly used indicators of PCAS neurological outcomes are the Cerebral Performance Categories (CPC) and Glasgow Outcome Scoring (GOS) systems, but these clinical scoring systems have limited value in the immediate hours and days following cardiac arrest^3^.

Electrophysiologic examination can offer a more accurate prediction of neurological outcomes at an earlier phase, but the requirement for expert interpretation may limit its widespread application^5^. Serum biomarkers such as neuron-specific enolase (NSE) and S-100B are easily obtained, but these indicators have limited value due to the absence of a verified threshold for identifying patients destined for a poor outcome^3^. Neuroimaging modalities for the evaluation of PCAS have been exclusively restricted to the anatomical level. At present, the use of computed tomography (CT) and magnetic resonance imaging (MRI) is largely limited to the exclusion of intracranial pathologies, such as hemorrhage or stroke^6^.

Positron emission tomography (PET) using fluorine 18 fluorodeoxyglucose (^18^F-FDG) is a molecular imaging technique that can evaluate cerebral glucose metabolism *in vivo*. The effect of cardiac arrest on brain glucose metabolic patterns has been reported in a limited number of clinical and preclinical PET studies^7–9^. These studies have consistently shown that cardiac arrest induces a global decrease in glucose metabolism. However, the regional vulnerability of brain metabolism to hypoxic-ischemic insults, rather than the reported global decrease, is almost certainly more important for predicting neurological outcomes of PCAS^10,11^. A recent study evaluated the regional distribution of glucose metabolism after hypoxic-ischemic insult caused by PCAS^12^. But the prognostic value of regional glucose metabolism vulnerability for predicting neurological outcomes has not been studied.

We hypothesized that the presence of region-specific alterations on the FDG brain PET would enable stratification based on brain damage, thus providing prognostic information for PCAS. In this preclinical study, we evaluated the distribution pattern of regional glucose metabolism immediately following cardiac arrest using a PCAS rat model. Then, the early distribution patterns were correlated with long-term neurological outcomes to demonstrate the feasibility of using FDG brain PET for the early prediction of neurological outcomes of PCAS. The ultimate goal of this preclinical study is to develop a PET-based prognostic index potentially applicable to clinical emergency.

## Methods

### Subjects

All experimental procedures were approved by the Institutional Animal Care and Use Committee of Catholic University Medical College. The approval number was CIMH2018-10. All experimental procedures were conducted in accordance with National Research Council guidelines for the care and use of laboratory animals (revised in 1996). All specific pathogen-free male Sprague Dawley rats (n = 18, 372.5 ± 23.68 g, 12-wk-old) were purchased from Daehan Bio Link (Eumseong, Korea) and were acclimated to a 12-hour light/dark cycle, 50–60% humidity, and given free access to food and water for 3 weeks before initiation of the experiment. For reporting of results, we complied with the Animal Research: Reporting *In Vivo* Experiments (ARRIVE) guidelines^13^.

### Experimental design

**Figure 1:**
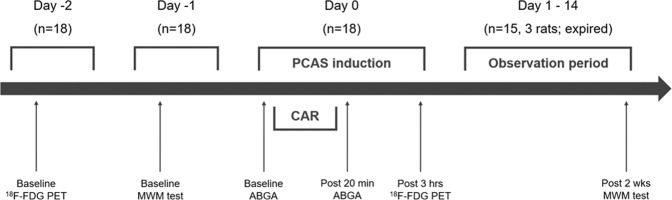
Flow chart of the study design. Prior to the post-cardiac arrest syndrome (PCAS) induction, all rats performed positron emission tomography (PET) scan and Morris water maze (MWM) test as baseline. During PCAS induction, arterial blood gas analysis (ABGA) was performed immediately before and 20-minute after the cardiac arrest and resuscitation (CAR) procedures. Three hours after CAR, rats were scanned with the same PET scanner. After 2-week of PCAS induction, rats were followed up with MWM test to evaluate neurological deficits.

The experimental design is summarized in Fig. 1. Briefly, all rats were assessed with PET scans (baseline) 2 days before induction of the PCAS model (day -2). The Morris water maze (MWM) test, a widely used neurocognitive test for evaluating learning and memory deficits in rodents, was performed 1 day before induction of the PCAS model (day -1)^14,15^. Three hours after induction, rats were scanned with the same PET scanner (post-3-hour scan) to investigate the prognostic value of ^18^F-FDG brain PET as an early indicator of neurological outcomes in a rat PCAS model. Two weeks after induction, rats were followed up with MWM test to evaluate the final neurological outcome (post-2-week MWM). According to the test results, rats were assigned to a good outcome group clinically corresponding to CPC 1 (good cerebral performance) or CPC 2 (moderate cerebral disability with independent activities) and a poor outcome group clinically corresponding to CPC 3 (severe cerebral disability) or higher. Then, the FDG brain PET results were compared between the two groups.

### Induction of the PCAS rat model

The post-cardiac arrest syndrome (PCAS) rat model was induced as described previously^12^. Anesthesia was induced with 14% isoflurane in an anesthesia chamber. Arterial blood gas analysis (ABGA) was performed immediately before model induction. Arterial blood samples for ABGA were collected from the femoral artery using sodium-heparin-coated syringes and analyzed using a GEM Premier 3500 (Instrumentation Laboratory, Lexington, MA). After the first ABGA, tracheal intubation was performed using an 18-gauge catheter. Animals were mechanically ventilated with a ventilator (R407, RWD Life Science, China) in volume control mode and 21% oxygen. Mean arterial blood pressure (MAP), heart rate, electrocardiography (ECG), and rectal temperature were continuously monitored with an IntelliVue MP40 GCX (Philips, USA). The right femoral artery was catheterized using polyethylene tubing (PE 50 mm I.D.) for blood sampling and continuous MAP monitoring. For ECG monitoring, two electrodes were placed subcutaneously on the chest. Rectal temperature was monitored and maintained at 36–37 °C using a temperature control system (Homeothermic Monitoring System, Harvard Apparatus, USA) throughout the whole experiment.

Anaesthetized rats were observed for 10 minutes following the surgical procedure. After observation, cardiac arrest was induced with a mixture of potassium chloride (JW Pharmaceutical, Korea). After the induction of cardiac arrest (MAP less than 10 mmHg), inhalation anesthesia and mechanical ventilation were stopped. Five minutes after cardiac arrest induction, cardiopulmonary resuscitation (CPR) was initiated with manual compression at a rate of 200 bpm, and mechanical ventilation was restarted with 100% fraction of inspired oxygen (FiO2). CPR quality was assessed by analyzing MAP waveforms. A single dose of calcium gluconate (15 ug/kg) (Daihan Pharm Co., Ltd., Korea) was intravenously injected. Then, a single dose of diluted epinephrine (5 ug/kg) (Daihan Pharm Co., Ltd., Korea) was intravenously injected every three minutes until return to spontaneous circulation (ROSC). A spontaneous MAP over 65 mmHg sustained for at least 30 seconds was considered as ROSC. CPR was terminated in rats that failed to achieve ROSC within 15 minutes, and these animals were excluded from the remainder of the experiment.

Mechanical ventilation was maintained after cardiac arrest until the end of the experiment. A second ABGA was performed 20 min after ROSC. Immediately after ABGA, the catheter was removed from the femoral artery, and the wound was closed. At 30 min after ROSC, FiO2 was decreased to 21%; 60 min after ROSC, the remaining monitors were removed, and the experiment was ended.

### Morris water maze test

An experienced investigator performed the behavioral testing of the animals using the modified method of previous study^16^. The maze consists of a circular pool (1.83 m diameter, 0.6 m depth) with a black interior filled with water maintained at a temperature of 22–24 °C. In the learning phase, a clear Plexiglas escape platform (10 cm diameter) was placed 1 cm below the surface of the water, rendering the platform invisible to the animal. The platform was placed in one of the quadrants and rats were trained to start finding for the hidden platform in other quadrants. Rats were trained with 4 trials/day for 5 consecutive days and given up to 120 seconds per trial to find the hidden platform. The testing room contained several prominent black and white cues visibly placed on the surrounding walls, which remained consistent throughout the testing period. A computerized tracking system (Accutrak®, San Diego, CA) was used to track and record animal movement and swimming patterns in the maze for all tests. Swim time and distance covered in the maze were recorded for each trial. After the completion of learning phase, rats were tested before and 2 weeks after the cardiac arrest and resuscitation procedures.

### ^18^F-FDG PET imaging

PET scanning was performed using a dedicated small animal PET system (microPET-R4; Concorde Microsystems, Knoxville, TN, USA). The spatial resolution at the center of field of view (FOV) was 1.3 mm. Rats were fasted for 12 hours (12.2 ± 0.9 hours) before each PET scan. Rats were anesthetized with 1.5% isofluorane, and radiotracers were injected intravenously (9.5 ± 0.7 MBq/0.1 ml). One hour after ^18^F-FDG injection, static brain PET images were acquired for 30 minutes. Rats were placed on a heating pad in a cage prior to PET scanning, and the temperature was maintained at 30 °C throughout the uptake period. The acquired images were reconstructed to 0.2 mm × 0.2 mm pixel size with 0.8 mm slice thickness using a three-dimensional ordered-subset expectation maximization (3D OSEM) algorithm.

### PET image analysis

PET data analysis was performed by an experienced nuclear medicine physician using PMOD 3.3 software (PMOD Technologies, Zurich, Switzerland). PET data obtained over 30 minutes were manually co-registered to a volume of interest (VOI) template for the rat brain (atlas provided in PMOD: “Px Rat (W. Schiffer)”). The mean standardized uptake value (SUV) was obtained for each VOI. The SUV was calculated according to the injected dose and the rat’s body weight. The left and right SUVs of paired structures were averaged. Subregions of the hippocampus and cerebellum were merged. Finally, VOIs of insular cortex, auditory cortex, cingulate cortex, frontal association cortex, medial prefrontal cortex, motor cortex, orbitofrontal cortex, parietal association cortex, retro-splenial cortex, somatosensory cortex, visual cortex, hippocampus, thalamus, midbrain, pons, medulla and whole brain were used for analysis. SUV change (SUV_delta_) of each region was calculated by subtracting post-3-hour SUV from baseline SUV. To evaluate relative glucose metabolism, the SUV ratio (SUVR) was obtained by dividing the SUV of each region with the SUV of the whole brain. The forebrain-to-hindbrain ratio (FHR) was calculated by dividing average of VOIs within forebrain regions with average of VOIs within hindbrain regions.

### Statistics

All statistical analyses were performed using the MedCalc software. Mann-Whitney tests were performed to analyze differences between two independent groups (good outcome vs. poor outcome), while Wilcoxon signed rank tests were performed to evaluate differences between two paired observations (baseline vs. post 3-hour). The cut-off between good and poor outcome group was derived from the median value of the swim time at the post-2-week MWM test. Receiver-operating-characteristic (ROC) curves were used to evaluate the predictive performance of PET uptake for the achievement of a good neurological outcome. The optimal cutoff value was determined using the Youden index, which is the value with the maximum sum of sensitivity and specificity. *P* values less than 0.05 were regarded as statistically significant. All data were presented as medians with interquartile ranges.

## Results

### Clinical course of the PCAS rat model

During the 2-week observation period, 3 rats died despite a successful CPR (1 rat; within 24 hour, 2 rats; within 48 hour). Therefore, the post-2-week MWM test was available for the remaining 15 PCAS rats. Post-2-week MWM swim time was significantly increased compared to baseline (10.1 sec, IQR 8.1–11.6 *vs*. 49.1 sec, IQR 41.3–86.7; *p* < 0.001). The post-2-week MWM swim distance was significantly greater than at baseline (202.9 cm, IQR 174.5–271.5 *vs*. 1651.9 cm, IQR 1237.9–2379.3; *p* < 0.001). The baseline and post-2-week MWM results for each rat are summarized in Supplemental Table 1.

**Figure 2:**
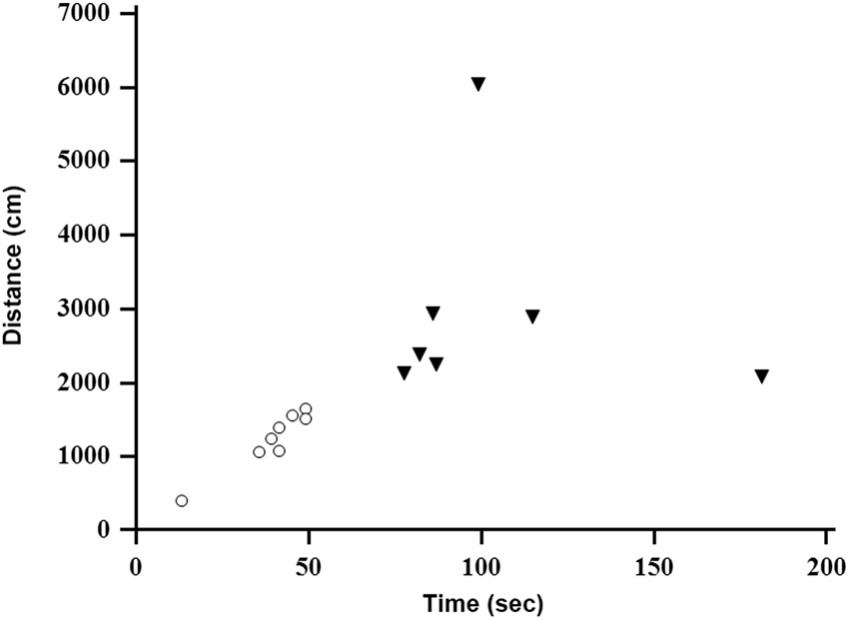
Scatter plot of Morris water maze (MWM) time and distance at the post-2-week time point. 15 PCAS rats are distributed by the post-2-week MWM swim time and distance. Eight rats (marked with circle) completed the maze in less than 50 seconds are assigned to the good neurological outcome group, while 7 rats (marked with triangle) more than 50 seconds are assigned to the poor neurological outcome group.

According to the post-2-week MWM results, 8 rats completing the maze in less than 50 seconds were assigned to the good neurological outcome group, while 7 rats with more than 50 seconds were assigned to the poor neurological outcome group. Distributions of PCAS rats by the post-2-week MWM results are shown in Fig. 2. The 3 rats that expired before the post-2-week MWM test were also assigned to the poor outcome group. Thus, a total of 10 rats were included in the poor outcome group. The pre- and post-experiment blood pressure, heart rate, and ABGA results were no significant differences in laboratory parameters between the good and poor neurological outcome groups post- or pre-experiment. Laboratory parameters are summarized in Supplemental Table 2.

### PET imaging analysis

#### Distribution of regional SUV in the baseline and post-3-hour PET scans

The baseline PET showed no significant difference in regional and whole brain SUV between good and poor outcome group (Supplemental Table 3). The post-3-hour PET showed a significant decrease of regional and whole brain SUV in poor outcome group compared with good outcome group except for medulla. The regional and whole brain SUV on the post-3-hour PET according to PCAS outcomes are summarized in Table 1.

**Table 1:** Regional and whole brain SUVs on post-3-hour PET according to PCAS outcome.

	Good outcome (n = 8)	Poor outcome (n = 10)	*p*
Insular Cortex	4.55 (4.03–5.35)	2.84 (2.46–3.85)	0.009*
Auditory Cortex	4.01 (3.75–5.54)	2.66 (2.27–3.43)	0.006*
Cingulate Cortex	5.24 (4.84–6.52)	3.22 (2.49–4.02)	0.004*
Frontal Association Cortex	4.73 (3.5–5.36)	2.43 (1.76–3.06)	0.001*
Medial Prefrontal Cortex	5.34 (5.15–6.36)	3.48 (2.67–4.61)	0.006*
Motor Cortex	4.84 (4.12–5.92)	2.67 (2.03–3.4)	0.002*
Orbitofrontal Cortex	5.22 (4.54–5.97)	3.12 (2.37–4.23)	0.002*
Parietal Association Cortex	4.41 (4.12–5.91)	2.6 (2.19–3.4)	0.006*
Retro-splenial Cortex	4.53 (3.83–5.46)	2.79 (2.32–3.54)	0.009*
Somatosensory Cortex	4.48 (4.23–5.9)	2.7 (2.25–3.56)	0.004*
Visual Cortex	4.55 (3.63–5.58)	2.66 (2.26–3.5)	0.009*
Hippocampus	3.78 (3.51–4.43)	2.65 (2.07–3.37)	0.004*
Thalamus	4.8 (4.47–5.84)	3.2 (2.43–4.27)	0.006*
Midbrain	4.36 (4.17–5.29)	3.07 (2.38–4.23)	0.016*
Pons	3.49 (2.84–4.3)	2.3 (1.82–3.42)	0.034*
Medulla	3.76 (3.06–4.67)	2.54 (2.13–3.86)	0.055
Whole Brain	5.94 (5.43–6.88)	3.02 (2.28–3.77)	0.001*

Values are shown as the median (interquartile range). Mann-Whitney test was used for statistical analysis. *p < 0.05.

#### SUV change (SUV_delta_) according to PCAS outcomes

The SUV_delta_ of each brain region was used to evaluate the decrease of glucose metabolism and showed regional difference between good and poor outcome group (Supplemental Fig. 1). The SUV_delta_ of the insular cortex, auditory cortex, cingulate cortex, medial prefrontal cortex, motor cortex, orbitofrontal cortex, parietal association cortex, retro-splenial cortex, somatosensory cortex, visual cortex, hippocampus and whole brain were significantly decreased in good outcome group compared with poor outcome group. The SUV_delta_ of the frontal association cortex, thalamus, and midbrain showed trends of decrease in good outcome group but not significant. However, the SUV_delta_ of the pons and medulla were not significantly different according to PCAS outcome (Table 2).

**Table 2:** Regional and whole brain SUV changes (SUV_delta_) between baseline PET and post 3-hour PET according to PCAS outcome.

	Good outcome (n = 8)	Poor outcome (n = 10)	*p*
Insular Cortex	1.33 (0.9–1.75)	2.46 (1.81–3.41)	0.034*
Auditory Cortex	1.46 (0.63–1.87)	2.56 (2.22–3.11)	0.012*
Cingulate Cortex	1.71 (0.64–2.79)	3.85 (2.86–4.47)	0.012*
Frontal Association Cortex	1.29 (0.08–1.89)	2.7 (1.61–3.16)	0.055
Medial Prefrontal Cortex	1.34 (0.36–2.53)	3.78 (2.37–4.41)	0.027*
Motor Cortex	1.15 (0.66–2.31)	3.49 (2.54–3.95)	0.016*
Orbitofrontal Cortex	1.28 (0.48–2.28)	3.3 (1.99–3.91)	0.043*
Parietal Association Cortex	1.48 (0.48–2.34)	3.27 (2.76–3.96)	0.012*
Retro-splenial Cortex	1.62 (0.18–2.44)	3.14 (2.35–3.92)	0.027*
Somatosensory Cortex	1.47 (0.77–2.24)	3.33 (2.56–3.82)	0.012*
Visual Cortex	1.42 (0.22–2.63)	3.17 (2.38–3.95)	0.027*
Hippocampus	0.76 (-0.08–2.16)	2.04 (1.33–2.61)	0.009*
Thalamus	1.39 (0.08–2.16)	2.89 (1.9–3.59)	0.055
Midbrain	1.17 (0.19–2.04)	2.5 (1.38–3.12)	0.083
Pons	1.17 (0.47–1.67)	1.71 (1.17–2.31)	0.101
Medulla	1.03 (-0.05–1.6)	1.56 (1.08–2.23)	0.101
Whole Brain	1.41 (0.39–1.73)	2.85 (1.85–3.44)	0.012*

SUV_delta_ = (baseline SUV) − (post-3-hour SUV). Values are shown as the median (interquartile range). Mann-Whitney test was used for statistical analysis. *p < 0.05.

#### Post-3-hour distribution of regional SUVR according to PCAS outcomes

The SUVR of each brain region normalized to the SUV of the whole brain was used to evaluate relative glucose metabolism on the post-3-hour PET and showed regional difference according to PCAS outcome at the post-2-week time point (Supplemental Fig. 2). The good outcome group showed significantly higher SUVR of the frontal association cortex, cingulate cortex, medial prefrontal cortex, motor cortex, orbitofrontal cortex, parietal association cortex, retro-splenial cortex, somatosensory cortex, and visual cortex compared with the poor outcome group. In contrast, the good outcome group showed significantly lower SUVR in the pons and medulla compared with the poor outcome group. The SUVR of the hippocampus, thalamus, and midbrain did not show a significant difference between the good and poor outcome group. All data are summarized in Table 3.

**Table 3:** Relative glucose metabolism (SUVR) on post-3-hour PET according to PCAS outcome.

	Good outcome (n = 8)	Poor outcome (n = 10)	*p*
Insular Cortex	0.99 (0.97–1.09)	0.99 (0.96–1.03)	0.829
Auditory Cortex	1 (0.91–1.07)	0.92 (0.88–0.99)	0.203
Cingulate Cortex	1.25 (1.15–1.3)	1.06 (0.98–1.1)	0.001*
Frontal Association Cortex	1.02 (0.88–1.11)	0.8 (0.66–0.92)	0.012*
Medial Prefrontal Cortex	1.24 (1.2–1.28)	1.16 (1.1–1.19)	0.001*
Motor Cortex	1.12 (1.04–1.18)	0.88 (0.8–0.97)	0.003*
Orbitofrontal Cortex	1.14 (1.1–1.22)	1.04 (0.96–1.11)	0.021*
Parietal Association Cortex	1.03 (1–1.21)	0.84 (0.79–0.95)	0.002*
Retro-splenial Cortex	1.02 (0.97–1.09)	0.87 (0.84–0.99)	0.021*
Somatosensory Cortex	1.08 (1.03–1.21)	0.92 (0.88–0.99)	0.006*
Visual Cortex	1.04 (0.96–1.14)	0.84 (0.81–0.99)	0.021*
Hippocampus	0.85 (0.81–0.89)	0.89 (0.84–0.92)	0.146
Thalamus	1.12 (1.05–1.17)	1.08 (1–1.13)	0.460
Midbrain	1.01 (0.94–1.05)	1.04 (1–1.12)	0.315
Pons	0.75 (0.62–0.84)	1.04 (0.75–1.1)	0.043*
Medulla	0.83 (0.65–0.92)	1.03 (0.88–1.19)	0.012*

SUVR = (SUV of each region)/(SUV of whole brain). Values are shown as the median (interquartile range). Mann-Whitney test was used for statistical analysis. *p < 0.05.

#### Predictive performance of the post-3-hour forebrain-to-hindbrain ratio for PCAS outcomes

**Figure 3:**
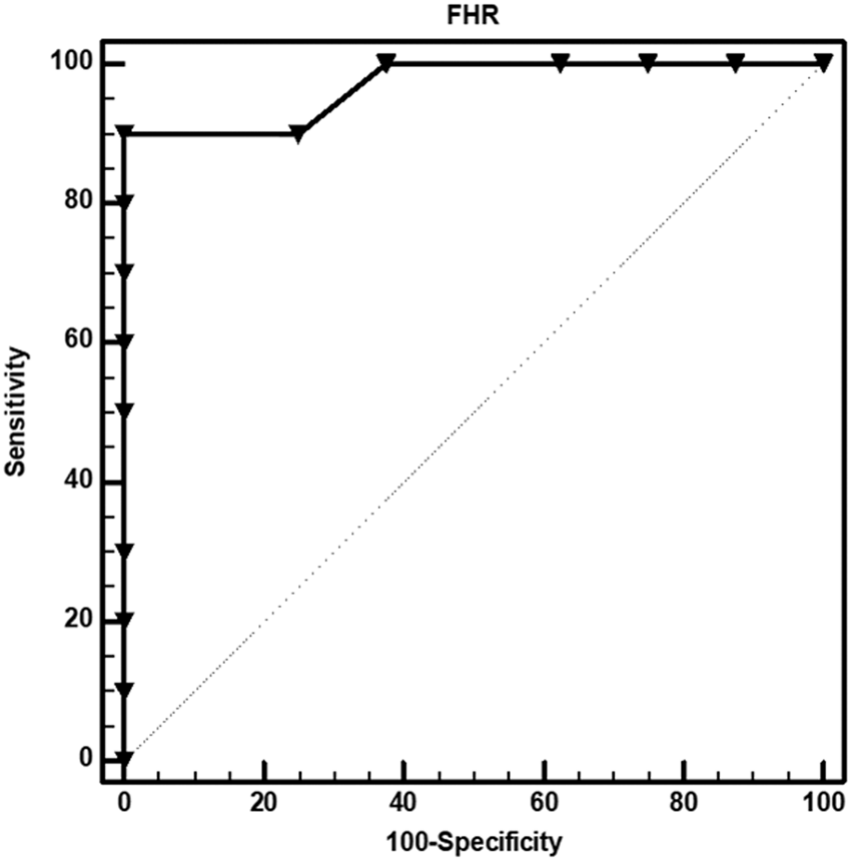
Predictive performance of the post-3-hour forebrain-to-hindbrain ratio for PCAS outcomes (ROC curves). Based on the differences in SUVR from the forebrain to hindbrain on the post-3-hour PET according to PCAS outcomes, forebrain-to-hindbrain ratio (FHR) was generated. With an optimal cutoff value of 1.22 (AUC 0.969, p < 0.001), FHR predicted good neurological outcomes with a sensitivity of 90% and specificity of 100%.

**Figure 4:**
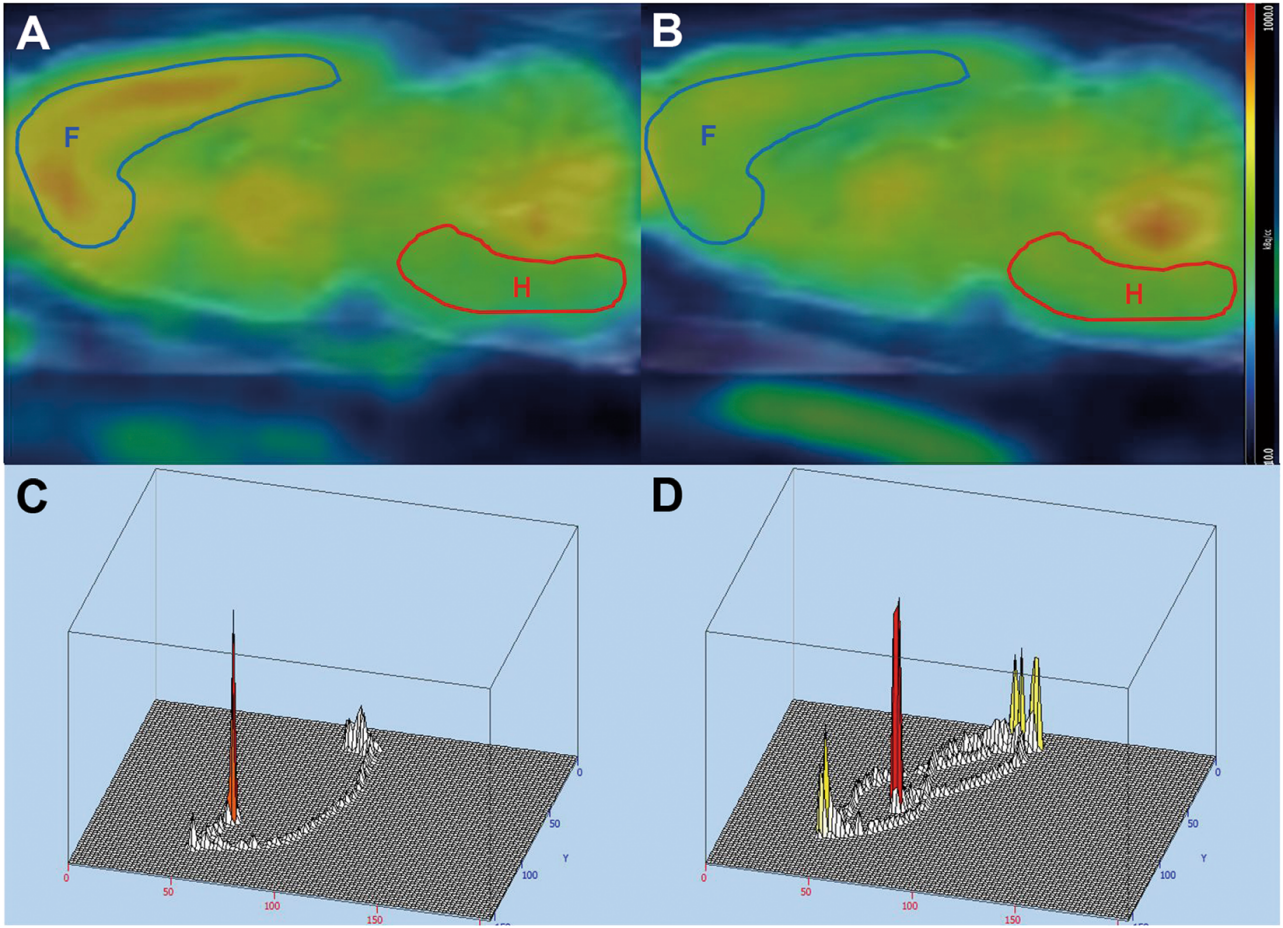
Representative cases of post-3-hour PET and Morris water maze test. On post-3-hour PET (**A**,**B**), the forebrain region (F) is marked with blue line, while the hindbrain region (H) is marked with red line. Left side presents the post-3-hour PET (**A**) and MWM data (**C**) of a good neurological outcome case. The post-3-hour PET revealed a forebrain-to-hindbrain ratio of 1.74. The maze time and distance recorded by a computerized tracking system were 13.1 minutes and 398.2 cm, respectively. Right side presents the post-3-hour PET (**B**) and MWM data (**D**) of a poor neurological outcome case. The post-3-hour PET revealed a forebrain-to-hindbrain ratio of 0.85. The recorded maze time and distance were 114.7 minutes and 2888.9 cm, respectively.

Based on the differences in relative glucose metabolism from the forebrain to hindbrain on the post-3-hour PET according to PCAS outcomes, we used ROC analyses to evaluate the predictive performance of the forebrain-to-hindbrain ratio (FHR) for the achievement of a good neurological outcome in Fig. 3. With an optimal cutoff value of 1.22 (AUC 0.969, *p* < 0.001), FHR predicted the achievement of a good neurological outcome with a sensitivity of 90% and specificity of 100%. Representative cases for the predictive value of FHR on the post-3-hour PET are described in Fig. 4.

## Discussion

PCAS has been well documented over the past three decades in emergency medicine departments and intensive care units since Negovsky first identified a distinct phase of resuscitation using specific pathology after ROSC^17^. The reduced hemodynamic state that accompanies PCAS can result in reversible or irreversible ischemic-hypoxic brain injury. Thus, therapeutic procedures for PCAS seek to recover normal cerebral functions and avoid neurological impairment. ^18^F-FDG PET can be used to evaluate metabolic and functional impairments in the brain after ischemic-hypoxic insults.

Schaafsma *et al*. investigated cerebral metabolic activity on ^18^F-FDG PET in resuscitated patients^8^. They reported a general decrease in glucose metabolism on day 1 post-resuscitation but did not describe any regional decreases for specific brain structures or vascular territories. Instead, they observed that regional differences followed patterns of neuronal density. Meanwhile, Choki *et al*. reported that the cerebral metabolic rate for glucose (CMRG) decreased to less than 50% of the control in the cerebral cortex while increasing to 200–300% in the hippocampus, globus pallidus and amygdala during the late stage of reperfusion in a stroke model^18^. A recent study by Putzu *et al*. using a PCAS model demonstrated a significant reduction in relative ^18^F-FDG uptake in the cortical areas, with an increase up to 33% in posterior regions such as the midbrain, pons, and cerebellum^12^. The authors suggested that the increase in glucose metabolism in these latter structures can be explained by the lower vulnerability of these structures to ischemia.

In this study, we also observed region-specific alterations in glucose metabolism for specific brain structures in a PCAS model. The post-3-hour PET showed a significant global reduction in brain SUV compared with the baseline PET. When the SUV of each brain region was normalized to the SUV of the whole brain to evaluate the relative glucose metabolism (SUVR), a difference in regional distribution between the baseline and post-3-hour PET was found. The SUVR of forebrain regions on the post-3-hour PET was significantly reduced compared with baseline, while the SUVR of the hippocampus, midbrain, and medulla on post-3-hour PET significantly increased, indicating a redistribution of energy consumption resulting from the lower metabolic vulnerability of these latter regions to ischemic insult.

The initial hypothesis of the current study was that the presence of region-specific alterations on the ^18^F-FDG PET would enable stratification based on brain damage, thus providing prognostic information for PCAS. The post-3-hour PET showed differences in the regional distribution according to final neurological outcome. The good outcome group showed a significantly higher SUVR in the forebrain regions along with a significantly lower SUVR of the hindbrain regions compared with the poor outcome group.

These data suggest that regional differences in SUVR on the post-3-hour PET are indicative of long-term outcomes in PCAS. A stepwise increase in metabolic vulnerability from the brain stem (least vulnerable) to the cortex (most vulnerable) may explain different outcomes in individuals with severe *vs*. mild neurologic impairment^19^. We also found that SUV_delta_ was indicative of outcomes in PCAS. However, the use of SUVR is more advantageous over SUV_delta_ for clinical emergency.

Numerous investigations have struggled to identify prognostic indicators for functional outcomes after ROSC, but no studies have found a reliable predictor^4^. Because the cost of highly intensive care for neurologically devastated survivors is a great burden on patients’ families and society at large, most studies have focused on identifying poor long-term prognoses based on clinical data or test findings of irreversible brain damage. Several systematics reviews have reported various predictors of poor outcome, including neurological examination, electrophysiological studies, biochemical markers, and neuroimaging^20^.

Brain CT is a widely used neuroimaging modality for PCAS in emergency settings. The major finding with PCAS is an attenuation of the gray-white matter interface, which is an indicator of cerebral edema and can be described as the gray-white matter ratio (GWR). Though most of these studies have recommended that the time interval between ROSC and CT not exceed 24 hours, no consensus currently exists regarding the optimal technique for quantifying GWR or the timing of CT acquisition for the prognostication of neurologic outcomes in PCAS. A recent study reported that generalized edema on brain CT predicted a poor neurological outcome with 97.6% specificity and 14.4% sensitivity within 24 h from ROSC^21^.

Brain MRI using diffusion-weighted (DW) imaging is considered the most sensitive and specific method for diagnosing and predicting PCAS. A restricted diffusion corresponding to areas of cytotoxic edema has been shown to occur as early as a few hours after the injury^22^. However, such a diffusion restriction may not be apparent with the global ischemic status in the first several hours of PCAS^23^. Moreover, MRI has limited utility in the least stable patients, and the optimal timing and approach for prognostic stratification remain controversial. Thus, current guidelines suggest using brain imaging studies for prognostication of PCAS only in combination with other clinical and electrophysiological predictors.

In this study, we performed ^18^F-FDG PET at 3 h after ROSC, which is early enough to inform treatment decisions for patients with PCAS. When the prognostic performance of the forebrain-to-hindbrain ratio (FHR) was evaluated as an early indicator of neurological outcome using ROC curves, 90% sensitivity and 100% specificity values were obtained with an optimal cutoff value of 1.22 (AUC 0.969, *p* < 0.05). Therefore, we speculate that FHR measured on post-3-hour PET is a useful indicator for the early prediction of neurological outcome in PCAS.

A small number of studies have investigated the prognostic value of brain ^18^F-FDG PET in resuscitated patients. Schaafsma *et al*. performed a prospective study in patients with PCAS to identify the prognostic value of regional vulnerability on PET. However, they acquired brain PET images on day 1 post-resuscitation, and because the study evaluated only a small cohort, all patients had a poor outcome. The comparison between survivors and non-survivors did not show any differences in PET data^8^. The clinical impact of the post-3-hour PET for predicting neurological outcomes in the post-resuscitation group has not been studied elsewhere. Future prospective studies in a larger patient cohort will be promising.

Our study has several limitations. First, we simply dichotomized the animals based on a final neurologic outcome of good or poor. Clinically, the most important point for patient stratification according to neurologic outcome is whether independent daily life is possible or not. However, it is difficult to apply the same criteria to rodents whether independent daily life is possible or not. Because the actual prognoses of PCAS patients vary to a much greater extent in the clinical setting, our experimental results should be interpreted cautiously. Nevertheless, the scatter plot of MWM time and distance at the post-2-week (Fig. 2) shows distinct distributions between groups. Second, the use of anesthesia in the experimental procedure may have affected the distribution of brain glucose metabolism, as anesthesia is known to reduce metabolism throughout the brain^12^. Nonetheless, animal anesthesia is necessary for obtaining *in vivo* PET imaging with an extended uptake time (in this study, 30 minutes). Because we conducted anesthesia under the same conditions for all rats, the main outcome of the current study will not be influenced by this experimental procedure. Third, corrections for blood glucose levels were not made in this study. Normalizing SUVs for individual blood glucose level could be an alternative technique as there are no optimal reference regions for normalization of ^18^F-FDG brain uptake in rodents^24^. Finally, the small sample size was somewhat insufficient for the statistical analysis, and thus these results may be affected by a certain degree of bias.

In conclusion, ^18^F-FDG brain PET has potential utility for the early prediction of neurocognitive outcomes in PCAS. In particular, a high forebrain-to-hindbrain ratio can be promising as a predictive indicator of good neurological outcomes.

## Supplementary information

Supplemental figure 1.

Supplemental figure 2.

Supplemental Table 1.

Supplemental Table 2.

Supplemental Table 3.

## Data Availability

All data generated or analyzed during this study are available from the corresponding author on reasonable request.
