# Supplementary figures and images for: Prognostic value of 18F-FDG brain PET as an early indicator of neurological outcomes in a rat model of post-cardiac arrest syndrome
Source: Sci Rep. 2019 Oct 15;9:14798. doi: 10.1038/s41598-019-51327-1 (PMC6794298; doi:10.1038/s41598-019-51327-1)

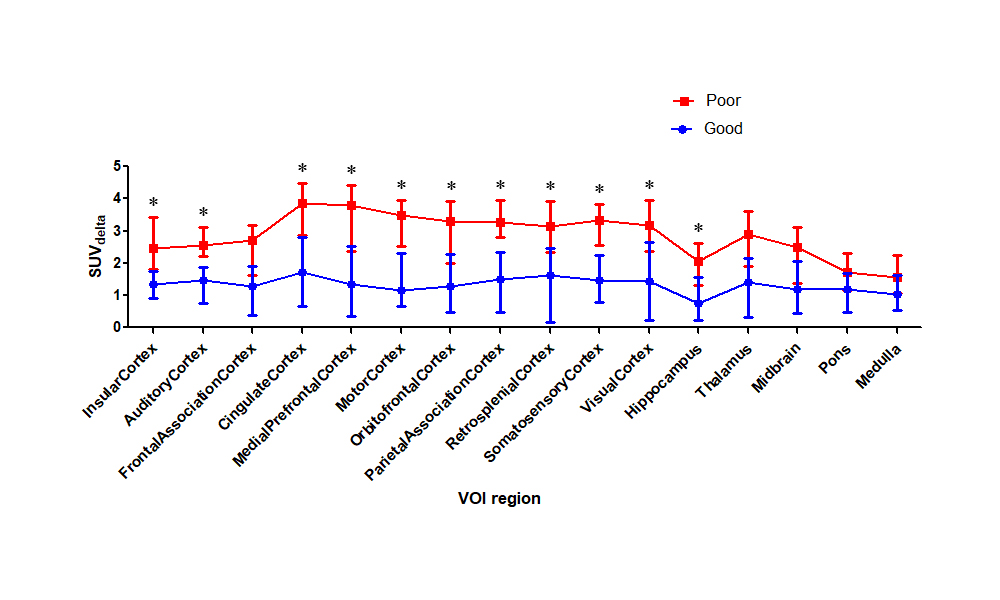

Supplement: Supplementary file 1 — Supplemental figure 1. [file 41598_2019_51327_MOESM1_ESM.docx]

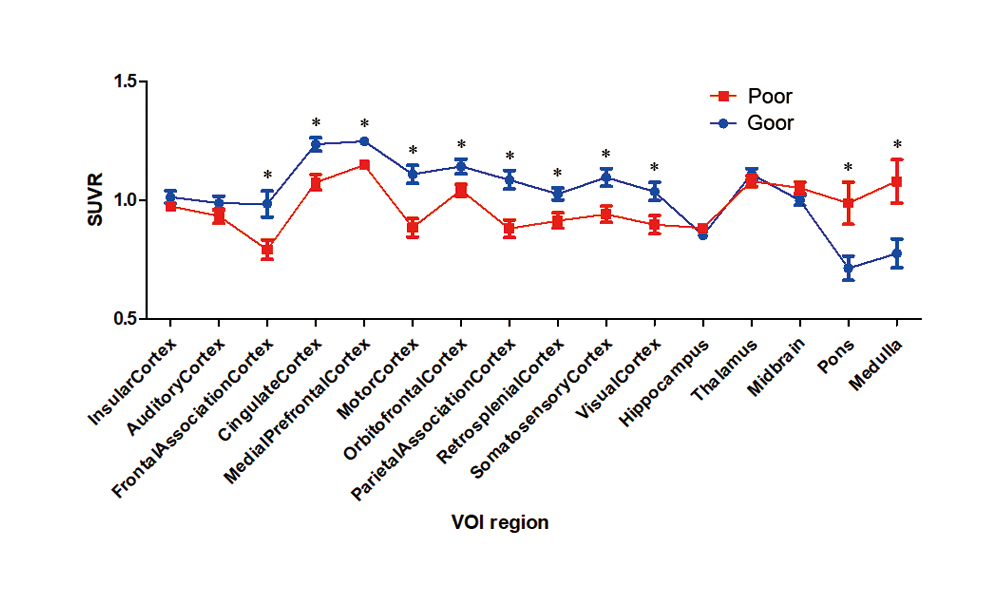

Supplement: Supplementary file 2 — Supplemental figure 2. [file 41598_2019_51327_MOESM2_ESM.docx]
