# Supplementary material for: Prognostic value of 18F-FDG brain PET as an early indicator of neurological outcomes in a rat model of post-cardiac arrest syndrome
Source: Sci Rep. 2019 Oct 15;9:14798. doi: 10.1038/s41598-019-51327-1 (PMC6794298; doi:10.1038/s41598-019-51327-1)
Supplement: Supplementary file 3 — Supplemental Table 1. [file 41598_2019_51327_MOESM3_ESM.docx]

**Supplemental Table 1.** **MWM time and distance of pre and post cardiac arrest for each animal**

| Subject | Pre-experiment | | | Post-experiment | |
| --- | --- | --- | --- | --- | --- |
|  | Time  (Seconds) | Distance  (cm) | Time  (Seconds) | | Distance  (cm) |
| No 1. | 8.8 | 26.2 | - | | - |
| No 2. | 9.9 | 297.7 | 82 | | 2379.3 |
| No 3. | 11.5 | 210.3 | - | | - |
| No 4. | 10.2 | 259.7 | 35.44 | | 1068 |
| No 5. | 6.7 | 119.4 | 77.44 | | 2129.6 |
| No 6. | 6.1 | 152.1 | 98.97 | | 6039.5 |
| No 7. | 11.5 | 215.4 | 13.1 | | 398.2 |
| No 8. | 12 | 285.5 | - | | - |
| No 9. | 6.31 | 175.4 | 41.25 | | 1081.9 |
| No 10. | 11.4 | 266.9 | 86 | | 2940.5 |
| No 11. | 6.4 | 171.9 | 45.25 | | 1564.3 |
| No 12. | 13.6 | 184.4 | 41.25 | | 1397.9 |
| No 13. | 10.4 | 197.3 | 86.75 | | 2241.6 |
| No 14. | 8.5 | 181.8 | 39.12 | | 1237.9 |
| No 15. | 9.4 | 194.6 | 49.13 | | 1651.9 |
| No 16. | 14.3 | 428.2 | 49 | | 1509.5 |
| No 17. | 9.5 | 384.2 | 114.69 | | 2888.9 |
| No 18. | 14.4 | 208.5 | 180.94 | | 2081.9 |

MWM: Morris water maze
