# Supplementary material for: Prognostic value of 18F-FDG brain PET as an early indicator of neurological outcomes in a rat model of post-cardiac arrest syndrome
Source: Sci Rep. 2019 Oct 15;9:14798. doi: 10.1038/s41598-019-51327-1 (PMC6794298; doi:10.1038/s41598-019-51327-1)
Supplement: Supplementary file 4 — Supplemental Table 2. [file 41598_2019_51327_MOESM4_ESM.docx]

**Supplemental Table 2. Comparison of laboratory measures between the good neurological** **outcome group and poor neurological outcome group**

|  | Pre-experiment | | | | Post-experiment | | |
| --- | --- | --- | --- | --- | --- | --- | --- |
|  | Good outcome (n=8) | Poor outcome (n=10) | *p* | Good outcome (n=8) | | Poor outcome (n=10) | *p* |
| SBP (mmHg) | 100.5 (96.8-107.5) | 100 (93.8-103.8) | 0.593 | 97 (72.3-103.5) | | 86.5 (71-100.5) | 0.091 |
| DBP (mmHg) | 59 (51.8-67) | 59 (55-64) | 1 | 56 (54.3-60) | | 60.5 (40-67.3) | 0.563 |
| HR (/min) | 297 (291-309.5) | 298 (288.5-308) | 1 | 299.5 (290-318.8) | | 297 (291.8-301.3) | 0.476 |
| pH | 7.37 (7.32-7.39) | 7.34 (7.31-7.39) | 0.655 | 7.28 (7.12-7.32) | | 7.31 (7.01-7.35) | 0.789 |
| PaO2 (mmHg) | 121.5 (103-129.8) | 108.5 (95.8-124.3) | 0.286 | 77.5 (69.5-102.5) | | 78 (66.3-92) | 0.533 |
| PaCO2 (mmHg) | 51.5 (50-57.3) | 55 (47.5-63) | 0.373 | 64.5 (58-78) | | 58 (50.3-81.5) | 0.327 |
| HCO3 (mmol/L) | 30.6 (26.9-32.6) | 27.9 (26.6-29.7) | 0.182 | 29.3 (27-32.2) | | 29.2 (21.6-33.7) | 0.965 |
| Base Excess (mmol/L) | 3.5 (0.9-4.4) | 1.4 (-0.25-2.75) | 0.11 | -0.1 (-4.2-3.6) | | 2.05 (-13.1-5.5) | 0.789 |
| SaO2 (%) | 99 (98.3-99) | 99 (97.5-99.3) | 0.723 | 93.5 (92.3-97) | | 95 (76.5-98) | 0.964 |
| Sodium (mmol/L) | 141.5 (140-142) | 140 (138.8-141) | 0.062 | 138.5 (138-140) | | 139 (137.8-141.3) | 0.685 |
| Potassium (mmol/L) | 4 (3.7-4.3) | 4.3 (3.8-4.6) | 0.166 | 4.7 (4.5-5.5) | | 4.6 (4.2-5) | 0.42 |
| Lactic Acid (mmol/L) | 4.2 (4.1-4.8) | 5.7 (4-6.3) | 0.228 | 6.6 (4.7-10.1) | | 5.4 (4.2-8.8) | 0.625 |

Values are expressed as the median (interquartile range).

SBP: Systolic Blood Pressure; DBP: Diastolic Blood Pressure; HR: Heart Rate
