# Supplementary material for: Prognostic value of 18F-FDG brain PET as an early indicator of neurological outcomes in a rat model of post-cardiac arrest syndrome
Source: Sci Rep. 2019 Oct 15;9:14798. doi: 10.1038/s41598-019-51327-1 (PMC6794298; doi:10.1038/s41598-019-51327-1)
Supplement: Supplementary file 5 — Supplemental Table 3. [file 41598_2019_51327_MOESM5_ESM.docx]

**Supplemental Table 3.** **Regional and whole brain SUVs on baseline PET scan according to PCAS outcome**

|  | Good outcome  (n=8) | Poor outcome  (n=10) | *p* |
| --- | --- | --- | --- |
| Insular Cortex | 5.78 (5.54-6.45) | 5.78 (5.53-5.93) | 0.696 |
| Auditory Cortex | 5.62 (5.19-6.41) | 5.59 (5.27-6.06) | 0.829 |
| Cingulate Cortex | 7.61 (6.95-7.78) | 7.01 (6.45-7.89) | 0.408 |
| Frontal Association Cortex | 6.24 (4.69-6.77) | 5.04 (4.1-5.51) | 0.101 |
| Medial Prefrontal Cortex | 7.01 (6.5-7.84) | 7.09 (6.78-7.96) | 0.696 |
| Motor Cortex | 6.83 (5.91-7.11) | 6.17 (5.51-6.67) | 0.274 |
| Orbitofrontal Cortex | 6.96 (5.87-7.29) | 6.31 (5.94-6.75) | 0.203 |
| Parietal Association Cortex | 6.47 (6.27-6.9) | 6.26 (5.78-6.91) | 0.360 |
| Retro-splenial Cortex | 6.16 (5.79-6.46) | 6.18 (5.58-7.01) | 0.897 |
| Somatosensory Cortex | 6.69 (6.16-7.06) | 6.18 (5.93-6.89) | 0.408 |
| Visual Cortex | 6.28 (5.74-6.45) | 6.2 (5.59-7.17) | 0.965 |
| Hippocampus | 4.66 (4.51-5.08) | 4.81 (4.61-5.06) | 0.460 |
| Thalamus | 6.54 (6.02-6.71) | 6.14 (5.67-6.69) | 0.460 |
| Midbrain | 5.82 (5.37-6.05) | 5.64 (5.24-6.07) | 0.573 |
| Pons | 4.53 (4.14-4.88) | 4.09 (3.87-4.75) | 0.173 |
| Medulla | 4.71 (4.49-4.98) | 4.36 (4.12-5.07) | 0.173 |
| Whole Brain | 5.94 (5.43-6.88) | 5.67 (5.22-6.28) | 0.573 |

Values are shown as the median (interquartile range).
